# Left main coronary artery morphological phenotypes and its hemodynamic properties

**DOI:** 10.1186/s12938-024-01205-3

**Published:** 2024-01-22

**Authors:** Qi Wang, Hua Ouyang, Lei Lv, Long Gui, Songran Yang, Ping Hua

**Affiliations:** 1 Department of Cardio-Vascular Surgery, Sun Yat-Sen Memorial Hospital, Sun Yat-Sen University, No. 107 Yan Jiang West Road, Guangzhou, 510120 China; 2 Department of Cardiovascular Surgery, Qilu Hospital of Shandong University, Shandong University, Jinan, China; 3 Department of Cardiac and Vascular Surgery, The First Affiliated Hospital of Kunming Medical University, Kunming Medical University, Kunming, China; 4 Department of Biobank and Bioinformatics, Sun Yat-Sen Memorial Hospital, Sun Yat-Sen University, No. 107 Yan Jiang West Road, Guangzhou, 510120 China

**Keywords:** Coronary artery, Cluster analysis, Hemodynamics, Atherosclerosis

## Abstract

**Background:**

Atherosclerosis may be linked to morphological defects that lead to variances in coronary artery hemodynamics. Few objective strategies exit at present for generalizing morphological phenotypes of coronary arteries in terms of hemodynamics. We used unsupervised clustering (UC) to classify the morphology of the left main coronary artery (LM) and looked at how hemodynamic distribution differed between phenotypes.

**Methods:**

In this study, 76 LMs were obtained from 76 patients. After LMs were reconstructed with coronary computed tomography angiography, centerlines were used to extract the geometric characteristics. Unsupervised clustering was carried out using these characteristics to identify distinct morphological phenotypes of LMs. The time-averaged wall shear stress (TAWSS) for each phenotype was investigated by means of computational fluid dynamics (CFD) analysis of the left coronary artery.

**Results:**

We identified four clusters (i.e., four phenotypes): Cluster 1 had a shorter stem and thinner branches (*n* = 26); Cluster 2 had a larger bifurcation angle (*n* = 10); Cluster 3 had an ostium at an angulation to the coronary sinus and a more curved stem, and thick branches (*n* = 10); and Cluster 4 had an ostium at an angulation to the coronary sinus and a flatter stem (*n* = 14). TAWSS features varied widely across phenotypes. Nodes with low TAWSS (L-TAWSS) were typically found around the branching points of the left anterior descending artery (LAD), particularly in Cluster 2.

**Conclusion:**

Our findings demonstrated that UC is a powerful technique for morphologically classifying LMs. Different LM phenotypes exhibited distinct hemodynamic characteristics in certain regions. This morphological clustering method could aid in identifying people at high risk for developing coronary atherosclerosis, hence facilitating early intervention.

**Supplementary Information:**

The online version contains supplementary material available at 10.1186/s12938-024-01205-3.

## Background

Atherosclerotic plaque formation within the coronary artery is a significant pathological feature of stenosis, and atherosclerosis is caused by a complex combination of inherited and environmental risk factors [1–3]. Despite the systemic nature of the risk factors that cause them, atherosclerotic plaques are not uniformly distributed and have unique locations, such as curvature and bifurcation [4, 5]. Computational fluid dynamics (CFD) is utilized to demonstrate how hemodynamic alter plaque vulnerability in coronary arteries. Atherosclerosis has been linked to low wall shear stress (WSS) and other hemodynamic abnormalities [6, 7]. Hemodynamic features are evidently influenced by the geometric characteristics of the arteries. Various studies have been conducted to evaluate the association between coronary geometry characteristics and lesion formation, which showed that geometry is closely related to histomorphometry even in normal coronary arteries by analyzing the area, length, curvature, angle of bifurcation, and other parameters of the coronary arteries and their daughter vessels [8–16]. The characteristics contributing to the atherosclerosis were named ‘geometric risk factors’ by Friedman et al. [9]. Whatever parameter is utilized to define the geometry, it must be simple to use, widely accepted by clinicians, and most importantly, efficiently describe the coronary artery characteristics and produce a uniform pattern.

Unsupervised clustering (UC), which can group samples based on a range of factors without specifying labels, could be useful in determining the best parameter. This strategy has been widely used in the field of cardiovascular diseases, such as the phenotypic grouping of patients with atrial fibrillation or heart failure, and it has been shown to improve disease phenotyping [10, 11]. We hypothesized that by combining UC with complex coronary morphological traits, we could overcome some of the limitations of conventional coronary morphological analysis methods and demonstrate how to use UC to classify vascular morphology phenotypes more effectively. Because the left main coronary artery (LM) supplies 84% of the blood flow to the left ventricle in individuals with right dominant circulation, lesions in the LM are the most serious coronary stenoses among all coronary diseases [12]. Therefore, we chose LM morphology as the clustering target and used CFD analysis to determine whether UC contributed to valid phenotyping of coronary arteries.

## Results

### Morphological characteristics of LM phenotypes

**Fig. 1:**
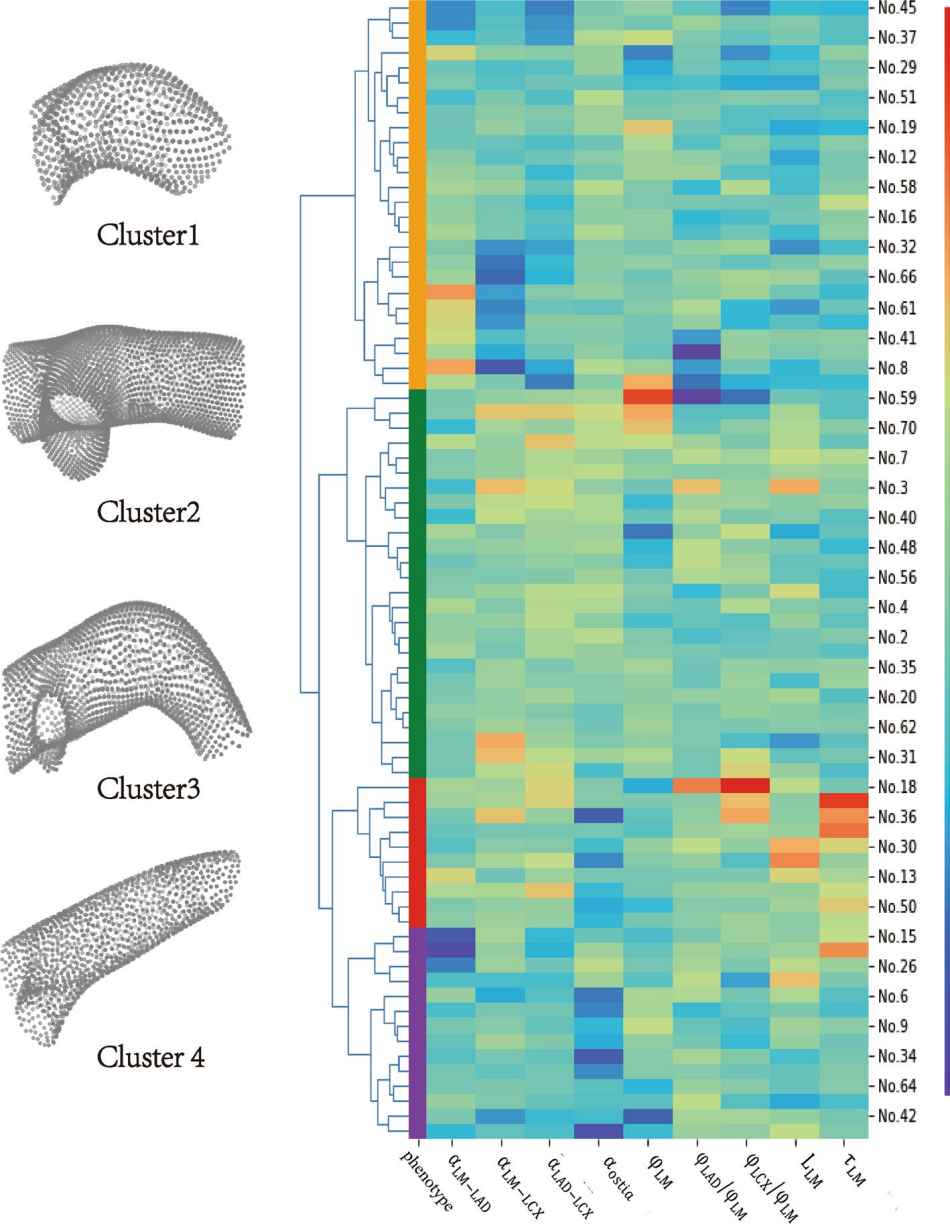
Morphological phenotypes of LM. The image shows representative left main (LM) coronary arteries from each of the four phenotypes, and hot plot describing clustering

A total of 158 patients who met the criteria were included in the study. After screening according to the exclusion criteria, 76 coronary arteries were eligible. Additional file 2: Results and Fig. S1 demonstrate the inclusion and exclusion of subjects. On the premise of ensuring the number of samples in each cluster, we could divide LM morphologies into 3 or 4 phenotypes by analyzing the cluster tree and characteristic heat map. Figure 1 displays the heat map of the clustering characteristics. By statistically analyzing the intergroup differences in geometric features, it could be found that the differences were more significant when divided into four phenotypes. Among them, the least cluster had 10 (13.16%) LMs and the most subgroup had 26 (34.21%). Additional file 2: Table S2 shows the clinical characteristics of the studied population and each phenotype.

Cluster 1 was the shortest phenotype, with a median $L_{{{\text{LM}}}}$ of the centerline only 6.33 mm (4.94–8.65 mm). The median $L_{{{\text{LM}}}}$ of the other three groups was around 10 mm, with significant statistical differences (6.33 mm vs. 9.83 mm, 11.66 mm and 10.82 mm, *p* < 0.001). The difference between Cluster 1 and other phenotypes was also reflected in the relative diameter of branches, with the median $\varphi_{{{\text{LAD}}}} /\varphi_{{{\text{LM}}}}$ being the thinnest at 0.78. Meanwhile, the $\varphi_{{{\text{LCX}}}} /\varphi_{{{\text{LM}}}}$ was also the thinnest, with a median diameter of 0.75 of LM. The angle of LCX emitted from LM was the straightest among the four phenotypes, with a median angle of $\alpha_{{{\text{LM}} - {\text{LAD}}}}$ only 46.76°, and the total bifurcation angle $\alpha_{{{\text{LAD}} - {\text{LCX}}}}$ was also the smallest. LM in Cluster 1 had a larger ostial angle, indicating that the ostia did not form a sharp angle with the aortic sinuses.

The ostia in Cluster 2 were similar to those in Cluster 1, with the largest ostial angle of four phenotypes. The median $\alpha_{{{\text{ostia}}}}$ was 80.06°, which was much greater than that of Cluster 3 and Cluster 4 (both with median angles less than 60°, *p* < 0.001). The median $\alpha_{{{\text{LAD}} - {\text{LCX}}}}$ of arteries in Cluster 2 exceeds 100°, which was significantly greater than that in Cluster 1 and Cluster 4.

Cluster 3 was the most twisty phenotype, with a median $\tau_{{{\text{LM}}}}$ exceeding 0.08, far exceeding the other three phenotypes. The LMs in this group were longer, reaching 11.66 mm (10.15–16.06 mm), but there was no statistically significant difference compared with Cluster 2 and 3. Different from the first two phenotypes, Cluster 3 had a smaller $\alpha_{{{\text{ostia}}}}$, indicating a larger angulation between the ostium and the aortic sinus.

As with Cluster 3, there was a larger angulation between the LM and the aortic sinus in Cluster 4. However, the $\tau_{{{\text{LM}}}}$ of the LMs in this phenotype was 0.03 (0.02–0.04), which was flatter than Cluster 3. Meanwhile, the LAD was thicker, with a median $\varphi_{{{\text{LAD}}}} /\varphi_{{{\text{LM}}}}$ of 89%. And the $\alpha_{{{\text{LM}} - {\text{LAD}}}}$ was the smallest among the four phenotypes, with a median angle of only 30.94°. This demonstrated that the LAD of this phenotype was more like a continuation of the LM.

**Fig. 2:**
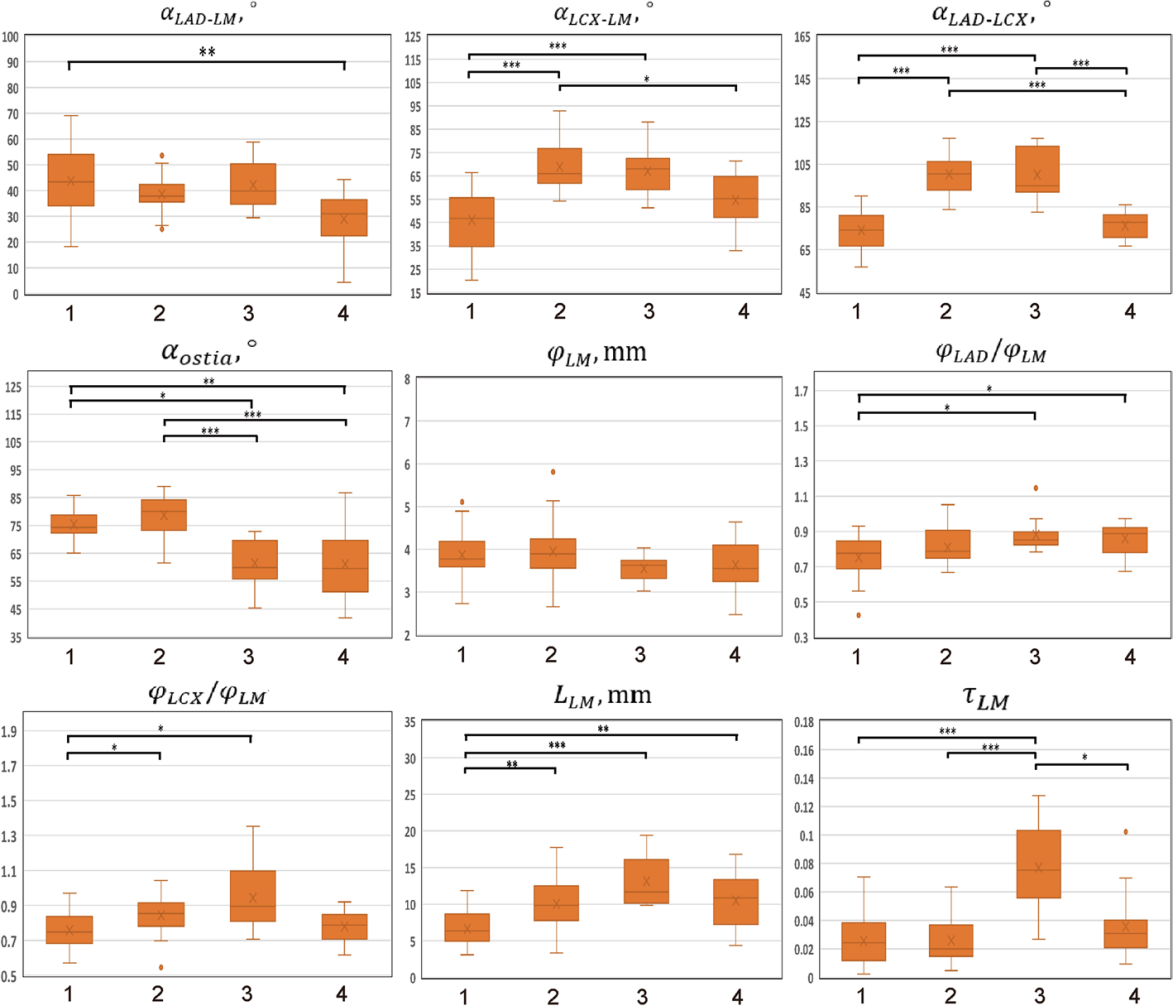
Geometric characteristics of 4 LM phenotypes. $\alpha_{{{\text{LAD}} - {\text{LM}}}}$, angle between left anterior descending artery (LAD) and left main (LM) coronary artery; $\alpha_{{{\text{LCX}} - {\text{LM}}}}$, angle between left circumflexus (LCX) and LM; $\alpha_{{{\text{LAD}} - {\text{LCX}}}}$, angle between LAD and LCX; $\alpha_{{{\text{ostia}}}}$, LM ostial title angle; $\varphi_{{{\text{LM}}}}$, the diameter of LM; $\varphi_{{{\text{LAD}}}} /\varphi_{{{\text{LM}}}}$, the ratio of LAD diameter to LM diameter; $\varphi_{{{\text{LCX}}}} /\varphi_{{{\text{LM}}}}$, the ratio of LCX diameter to LM diameter; $L_{{{\text{LM}}}}$, arc length of LM; $\tau_{{{\text{LM}}}}$, tortuosity of LM; *p < 0.05; **p < 0.01; ***p < 0.001

By analyzing the morphological characteristics of each LM phenotype, they could be summarized into Cluster 1 with a short stem and thin branches; Cluster 2 with a large bifurcation angle; Cluster 3 with an ostium at an angulation to the coronary sinus and a twisty stem with thick branches; and Cluster 4 with an ostium at an angulation to the coronary sinus but a straight stem. The detailed morphological characteristics of each subgroup are shown in Additional file 1: Table S1 and Fig. 2.

### Spatial variations of L-TAWSS in different phenotypes

The CFD nodes of the LM and the reserved bifurcation segments were downsampled to 700, and a total of 53,200 sampling CFD nodes were obtained. A total of 51,023 CFD sampling points were included by screening out extreme outliers of the TAWSS values. Among them, L-TAWSS accounted for 2.7%, with a total of 1361.

**Fig. 3:**
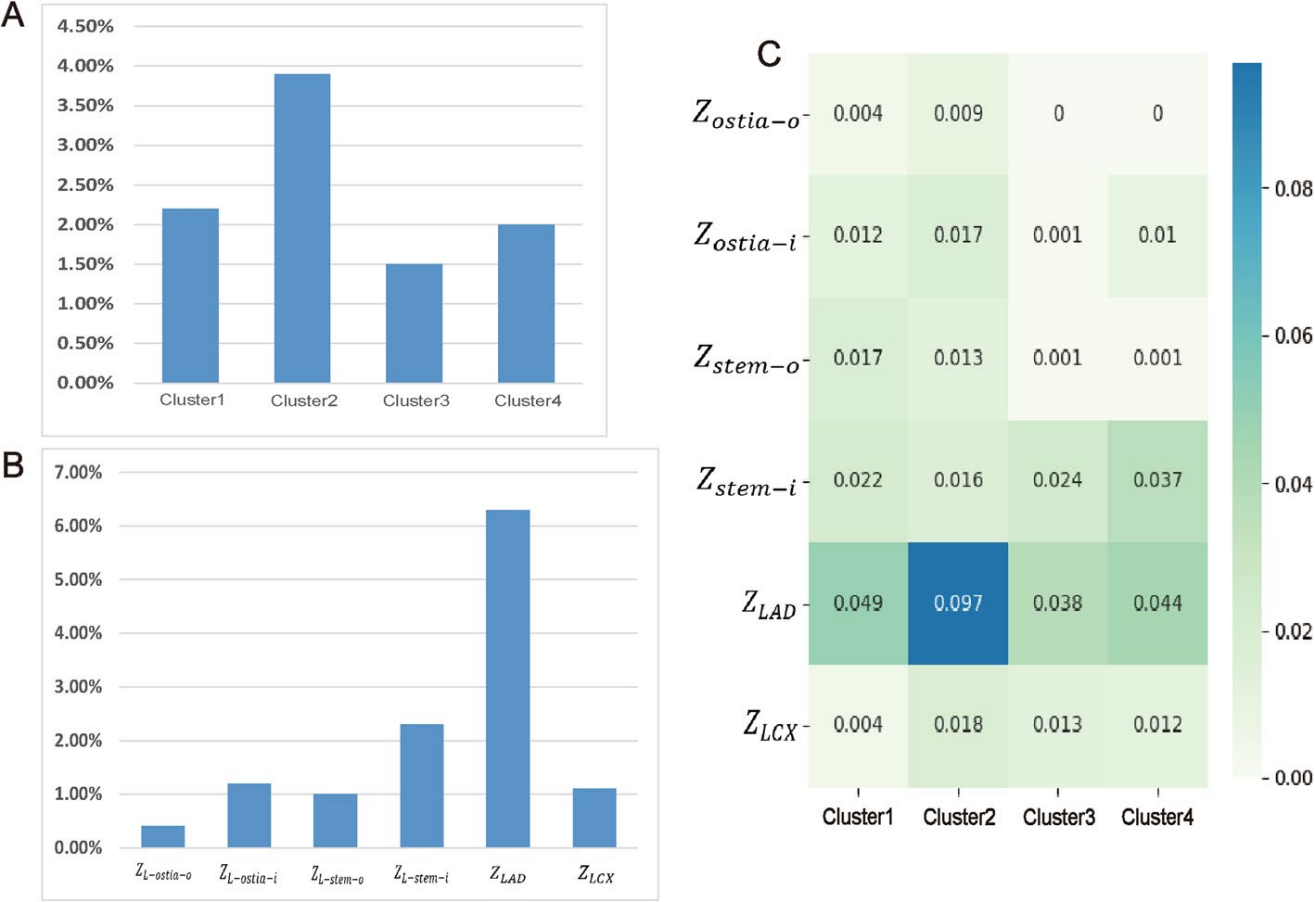
Distribution of L-TAWSS nodes. **A** The proportion of low time-averaged wall shear stress (L-TAWSS) nodes in different phenotypes. **B** The proportion of L-TAWSS nodes in different regions. **C** The heatmap showing the proportion of L-TAWSS in different regions for each phenotype

There were significant differences in the distribution of L-TAWSS among different phenotypes (*p* < 0.001). Cluster 2 is the grouping with the highest presence of L-TAWSS, with a proportion of 3.9%, significantly higher than the 1.50% of Cluster 3. The proportion of L-TAWSS in Cluster 1 and 4 was between the above two phenotypes. Figure 3A demonstrates the distribution of L-TAWSS in different phenotypes.

From the perspective of each individual region (Fig. 3B), the L-TAWSS is most commonly found in the ${\rm Z}_{{{\text{LAD}}}}$, followed by the ${\rm Z}_{{{\text{stem}} - i}}$, and is least commonly found in the ${\rm Z}_{{{\text{ostia}} - o}}$.

Through detailed partitioning, it could be found that there were similarities and significant differences between different regions of different morphological phenotypes (Fig. 3C). The high-risk L-TAWSS nodes were rarely distributed at ${\rm Z}_{{{\text{ostia}} - o}}$, especially in Cluster 3 and 4, where all nodes were not L-TAWSS.

There were more high-risk nodes in the stem segment far from the ostium. The increase of L-TAWSS was mainly in Cluster 1 and 2 at ${\rm Z}_{{{\text{stem}} - o}}$, while the increase was more obvious in Cluster 3 and 4 at ${\rm Z}_{{{\text{stem}} - i}}$. Similar to ${\rm Z}_{{{\text{ostia}} - o}}$, Cluster 3 and 4 had few L-TAWSS nodes at ${\rm Z}_{{{\text{stem}} - o}}$. However, Cluster 4 had a significant increase at ${\rm Z}_{{{\text{stem}} - i}}$, with a percentage of 3.7%.

At the bifurcation, the distribution of L-TAWSS was different in each phenotype. The increase in high-risk sites was mainly manifested at the ${\rm Z}_{{{\text{LAD}}}}$, while it was not evident at the ${\rm Z}_{{{\text{LCX}}}}$. This phenomenon was noticeable in Cluster 2, which had a significantly higher proportion of L-TAWSS at ${\rm Z}_{{{\text{LAD}}}}$ of 9.7% than the other phenotypes. Meanwhile its proportion at ${\rm Z}_{{{\text{LCX}}}}$ was only 0.4%, which was significantly lower than the other three clusters.

**Fig. 4:**
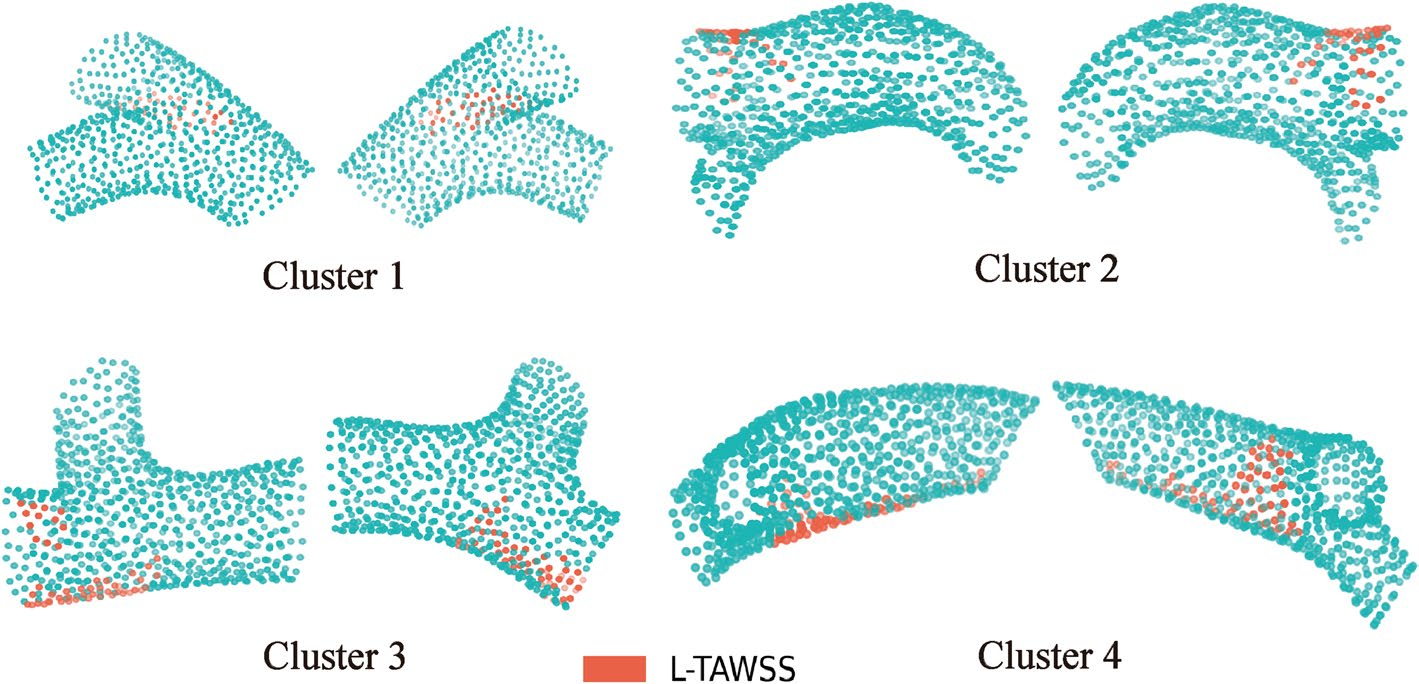
Example of the L-TAWSS nodes distribution for each cluster. L-TAWSS, low time-averaged wall shear stress

Overall, the high-risk nodes in Cluster 1 were more scattered and distributed in most of the regions except for the outer curve of the ostial area and LCX. The proportion of L-TAWSS in Cluster 2 was the highest among the four phenotypes and was widely distributed, with the most pronounced at the LAD. Cluster 3 had the lowest proportion of L-TAWSS, which was mainly distributed in the bifurcation and inner curve of the stem. While no differences in overall L-TAWSS proportions were observed between Cluster 1 and 4, there were variations between partitions. The distribution of L-TAWSS in Cluster 4 was similar to that of Cluster 3. Figure 4 shows an example of the L-TAWSS nodes distribution for each cluster.

## Discussion

This study used 76 authentic left coronary arteries and its branches, segmenting the LM and partitioning it using the vascular centerline and the Frenet–Serret frame. We used a UC-based cluster analysis on arterial morphology and identified four distinct phenotypes. Significant differences in hemodynamic characteristics were found among LM clusters using CFD analysis of all arteries. To precisely define this differential profile, we segmented the LM into 6 additional intergroup-consistent regions. Using this method, an LM with a realistic overall morphology was created, and it was discovered that different phenotypes had distinct high-risk regions that may be linked to the development of coronary atherosclerotic plaques. There were both consistency and heterogeneity in TAWSS between morphological phenotypes and different partitions. These variations can be exploited to account for the dispersed distribution of coronary atherosclerotic plaque and to identify regions at high risk for plaque formation in different LM morphological phenotypes. Additional information for clinical intervention may be gleaned from refining this uniform pattern of geometric phenotypes.

It is not a novel idea that plaque formation in the coronary arteries may be affected by certain geometric characteristics. In 1983, Friedman et al. introduced the term ‘geometric risk factors’ for atherosclerosis. Artery length [17], angle of bifurcation [5], curvature [18], and cross-sectional [13] have all been studied for their role in plaque development, and there is also considerable debate regarding LM length [19, 20]. However, hemodynamics is affected by a number of geometric risk factors, rendering it impracticable to precisely evaluate using a solitary morphological parameter. The need for analysis of geometric characteristics and hemodynamics has grown in recent years as the significance of interactions between multiple geometric features and blood flow have been appreciated by more and more scientists. In order to characterize UC traits, we used a wide variety of geometric metrics connected to LMs in this study. To the best of our knowledge, this is the first study to apply UC to the study of LM phenotypes. We discovered both homogeneity and heterogeneity among the three LM phenotypes we identified, as measured by the selected geometric parameters, such as arc length, bifurcation angle, ostial angle, and tortuosity. As these phenotypes were developed using data from a real population, they have the potential to generalize typical LM geometries and are interpretable, thereby enhancing their clinical utility in the detection of atherosclerotic forms with a high risk of progression [21].

It is widely accepted that intrinsic plaque formation factors include hemodynamic variations resulting from morphological defects. TAWSS is one of the most studied topics [14, 22]. WSS is a tangential force generated and exerted on the endothelium surface due to blood flow friction [14, 18]. Adequate WSS is necessary for maintaining cardiovascular function and protecting arteries, whereas low WSS contributes to the formation of atherosclerosis [7, 14, 18, 23, 24]. According to previous studies, nodes ≤ 0.4 Pa were defined as low TAWSS, i.e., L-TAWSS. As for spatial variations, we partitioned LMs into six geometrically consistent regions and identified high-risk regions for each LM phenotype by evaluating the distribution of CFD nodes within each region [6].

WSS was generally higher in the ostium of the LM than in other regions, where the likelihood of atherosclerosis formation was also lowest. The outer curve of the LM was subject to a greater velocity gradient due to the impact of blood flow, so that the L-TAWSS was significantly less than the inner side. This result was consistent with Chiastra et al.’s study that inner side of arteries is more prone to atherogenesis [18, 25]. Cluster 1 had the smallest angle between LCX and LM, and its L-TAWSS accounted for less. In contrast, Cluster 2 had the most high-risk nodes, mainly due to its large $\alpha_{{{\text{LM}} - {\text{LCX}}}}$ and $\alpha_{{{\text{LAD}} - {\text{LCX}}}}$. The percentage of L-TAWSS at its bifurcation was the highest in all partitions of all phenotypes, and mainly distributed on the LAD. This phenomenon was in line with previous findings that arterial flow disturbances were exacerbated in arteries with large bifurcation angles, leading to low TAWSS [26, 27], thus reinforcing the blood flow environment in which atherosclerosis occurs [28]. Friedman et al. found that when a daughter vessel divides from the main stem at a larger angle, the other daughter vessel that is contiguous to the main stem is more prone to low WSS, which is one of the classical ‘geometric risk factors’ [9].

In addition, we found some discrepancies in the results of prior studies. In the analysis section of LM morphology, we suspected that the inner curve of the stem in Cluster 3 had a higher risk of atherogenesis based on geometric risk factors, which were based on previous studies about the relationship between the curvature and atherosclerosis [25]. However, the simulation of TAWSS nodes revealed that Cluster 3 did not have an outstanding number of L-TAWSS at ${\rm Z}_{{{\text{stem}} - i}}$, even less than other phenotypes with flatter stems. In the same way, this cluster had the least L-TAWSS at ${\rm Z}_{{{\text{ostia}} - i}}$. Morphological analysis revealed that while the stem of Cluster 3 was significantly curved, there was a noticeable angulation between its ostium and the coronary sinus, which resulted in the direction of blood flow at the entrance not being parallel to the long axis of the stem. Compared to the phenotype with a straight LM, the former was more compliant with the direction of blood flow, resulting in fewer L-TAWSS nodes as the blood flow through here did not lose momentum and form a vortex. This finding highlighted the limitations of previous studies by demonstrating how difficult it is to define coronary geometry and how evaluating the vessel’s hemodynamic characteristics based on a single geometric aspect may be inaccurate. In this study, the UC method was used to incorporate a number of morphological features without resorting to manual combining. The newly generated phenotypes integrated the essential features of each, making them more representative of reality. Our findings shed new light on the morphology-based coronary artery classification approach, which holds promise for its potential clinical application in identifying high-risk individuals for coronary heart disease.

## Limitations

Our findings are limited by a few caveats. Because there were insufficient RM-positive LMs for reliable clustering, we were forced to exclude them from the analysis. Clustering is unsupervised, but it still requires human oversight to ensure that the appropriate characteristics are considered. It is crucial to keep in mind that data play a central role in clustering and that the outcomes can be altered by selecting different characteristics or populations [29]. In addition, our study incorporated patients without the inclusion of invasive blood pressure monitoring or catheterization to measure coronary flow velocity. Consequently, the utilization of patient-specific flow curves was precluded in CFD analysis. Finally, none of the participants exhibited newly developed coronary atherosclerosis within the time period of our present study. In order to conclusively demonstrate the significance of morphological clustering, our future research should incorporate a clinical follow-up.

## Conclusion

Our findings suggest that UC can be employed for morphological classification of coronary arteries. Hemodynamic abnormalities resulting from these distinct phenotypes have been linked to the development of coronary atherosclerotic plaques, according to our CFD findings. This approach evaluates the influence of various characteristics comprehensively, which is closer to the actual scenario. Our findings may serve as a proof of concept that UC can be employed for morphological phenotyping of coronary arteries and may assist in identification of patients at high risk for developing coronary atherosclerosis.

## Methods

The method used in this study is depicted below, and the flowchart can be found in Additional file 2: Fig. S2.

### Study population

Patients who underwent coronary computed tomography angiography (CCTA) at Sun Yat-sen Memorial Hospital, affiliated with Sun Yat-sen University, between March 2019 and May 2019 on the suspicion of CAD but who did not have detectable coronary atherosclerotic plaques on CCTA, were selected retrospectively. Patients were screened out if they had structural heart disease, a history of cardiac surgery, severe coronary myocardial bridge, poor image quality, or coronary artery malformations. We also excluded arteries with ramus intermedius artery (RI) from our analysis due to the small sample size and the possibility that RI could have a major effect on hemodynamics.

### Coronary reconstruction and sectioning in 3D

Each slice in the CCTA sequence is 0.6 mm thick. All images were acquired at the end of diastolic phases (typically 70–80 percent of the RR interval, according to the heart rate). To segment the 3D coronary artery lumen mask and determine the centerline, the original digital DICOM data were imported into Mimics (version 21.0, Materialise Software, Wilfried, Leuven, Belgium) [13]. All left coronary arteries and its branches were segmented and reconstructed from the coronary ostia to the distal left anterior descending (LAD) and left circumflexus (LCX) with major side branches. We followed Frank Gijsen’s proposal to evaluate the survival of branches and distal vessels [14]. We also got rid of inferior-quality branches.

### Geometric measurements and morphological clustering

**Fig. 5:**
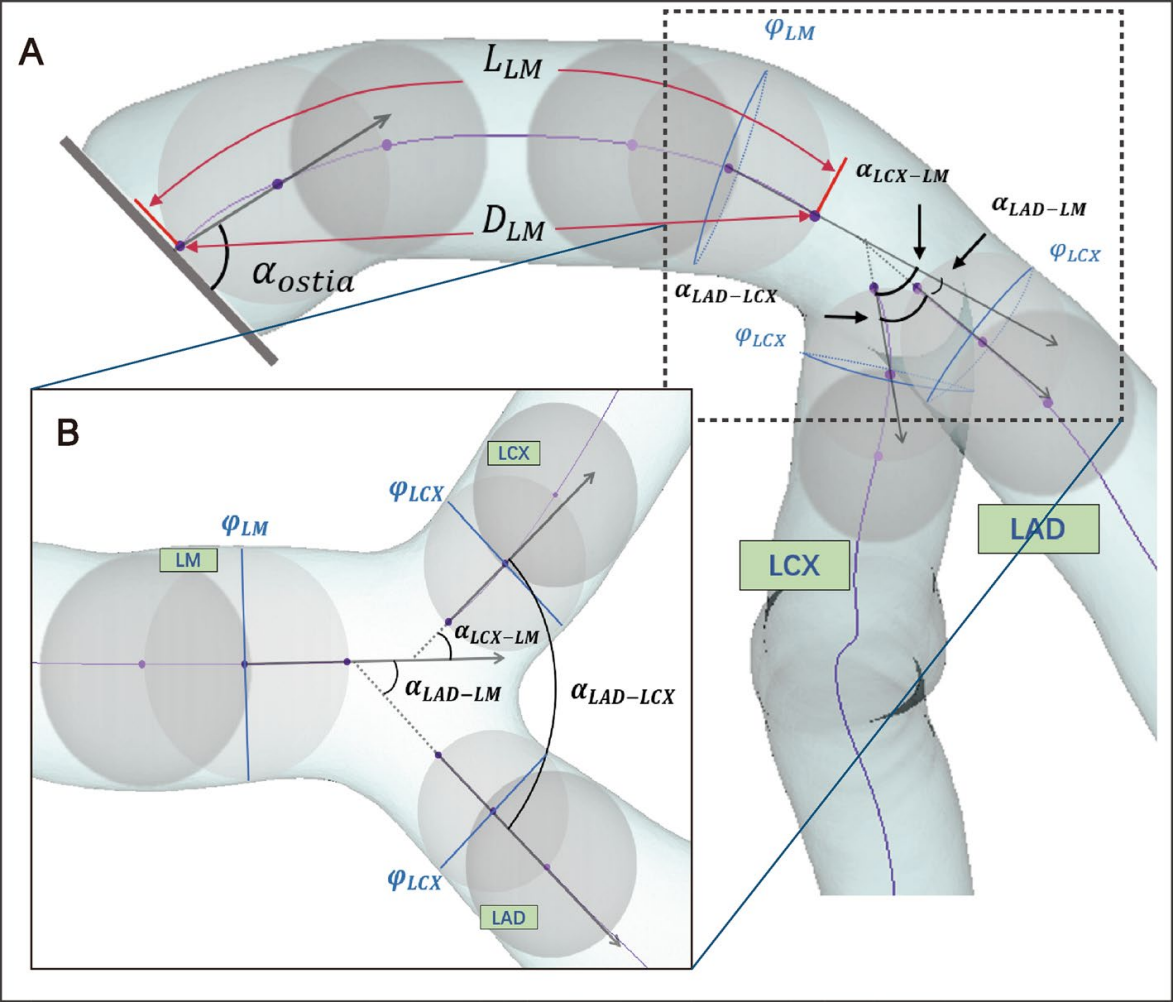
Description of geometric parameters of LM. *LAD* left anterior descending artery, *LCX* left circumflexus, *LM* left main coronary artery, *RCA* right coronary artery, $D_{{{\text{LM}}}}$, distance between the endpoints of LM; $L_{{{\text{LM}}}}$, arc length of LM; $\alpha_{{{\text{LAD}} - {\text{LCX}}}}$, angle between LAD and LCX; $\alpha_{{{\text{LAD}} - {\text{LM}}}}$, angle between LAD and LM; $\alpha_{{{\text{LCX}} - {\text{LM}}}}$, angle between LCX and LM; $\varphi_{{{\text{LM}}}}$, inscribing circle diameter of distal LM; $\alpha_{{{\text{ostia}}}}$, LM ostial title angle; $\varphi_{{{\text{LAD}}}}$, inscribing circle diameter of proximal LAD; $\varphi_{{{\text{LCX}}}}$, inscribing circle diameter of proximal LCX

The geometric characteristics at LM and bifurcations were calculated by Mimics and Python (version 3.8, with matplotlib and Scipy packages). We established more stringent and objective criteria for the measurement of geometric parameters to avoid operator bias, standardizing the qualities required for morphological clustering analysis [15]. An inscribing circle could be fitted based on the cross-section of the artery by using the control point on the centerline as the circle’s center. The first control point was designated as the first center of the circle, from which the subsequent inscribing circle was constructed. The control point in closest proximity to the inscribing circle was selected as the center for the second circle, so enabling the creation of the second inscribing circle. The arterial branches’ starting points and angle measurement positions were combined based on the inscribing circle’s position and diameter. We established 3 vectors in the direction of blood flow using the first and second circle centers at the distal LM and proximal LAD/LCX as endpoints, and then measured the angles between the vectors separately (Fig. 5).

The parameters measured for each patient were as follows:

- Angles of the bifurcation ($\alpha_{{{\text{LCX}} - {\text{LM}}}} , \;\alpha_{{{\text{LAD}} - {\text{LM}}}} ,\;\alpha_{{{\text{LAD}} - {\text{LCX}}}}$) (Fig. 5A, B)
- LM ostial title angle ($\alpha_{{{\text{ostia}}}}$) (Fig. 5A)
- Inscribing circle diameter of the coronary adjacent to the bifurcation ($\varphi_{{{\text{LM}}}}$, $\varphi_{{{\text{LAD}}}}$, $\varphi_{{{\text{LCX}}}}$) (Fig. 5A, B)
- Arc length of LM ($L_{{{\text{LM}}}}$) (Fig. 5A)
- Straight-line length between the endpoints of LM ($D_{{{\text{LM}}}}$) (Fig. 5A)
- Tortuosity of LM ($\tau_{{{\text{LM}}}}$)

The definition of tortuosity is as follows [16]:

1 $$ \begin{array}{*{20}c} {\tau_{{{\text{LM}}}} = 1 - \frac{{D_{{{\text{LM}}}} }}{{L_{{{\text{LM}}}} }}} \\ \end{array} . $$

After normalizing for outliers, morphological clustering is done on the aforementioned characteristics. We categorized morphological characteristics into hierarchical clusters using Python (version 3.8) and matplotlib, statsmodels, and seaborn packages. The clusters were identified with the aid of the Ward minimum variance technique. These tasks were completed independently by two experienced clinicians.

### CFD analysis of LMs

The reconstructed arteries were then smoothed and refined. We employed tetrahedral meshes and four boundary-fitted prism layers for each scenario. Blood flow was defined by fluid domains, while the inlet, outlet, and wall of the cropped vessel were defined by solid domains. In each instance, the coronary ostium was considered an inlet and the distal side branches of cropped arteries were considered an outlet.

A transient analysis was chosen during the CFD computation procedure. The flow rate curve at the inlet and the pressure curve at the outlet were derived from literature due to the absence of patient-specific flow data. Blood, with a density of 1060 kg/m^3^, was regarded as a non-Newtonian fluid, and the Carreau model was employed to simulate the viscosity of blood, which proved to be more accurate in coronary arteries [13, 30, 31]. And it was supposed that the artery walls were stiff and non-slippery. Since the Reynold number is less than 750, it was believed that the transient model was laminar. We set the cardiac cycle to 0.8 s, calculated three cardiac cycles for each case, and output the time-averaged wall shear stress (TAWSS) for the last cycle as a result of post-processing. Convergence was defined as a residence error with a root mean square of less than 10^–6^ for each time step. Additional details are contained in Additional file 2: Method.

After extracting the CFD results of LM segments from each case, all nodes of each retrieved arteries were downsampled to 700 nodes using farthest point sampling in an effort to enhance computational efficiency and ensure accuracy [32]. Based on previous studies [14, 33], nodes with TAWSS < 0.4 Pa were defined as L-TAWSS, which were regarded as high-risk nodes that were more prone to atherosclerosis. Tukey’s test was used to filter and remove extreme outliers.

**Fig. 6:**
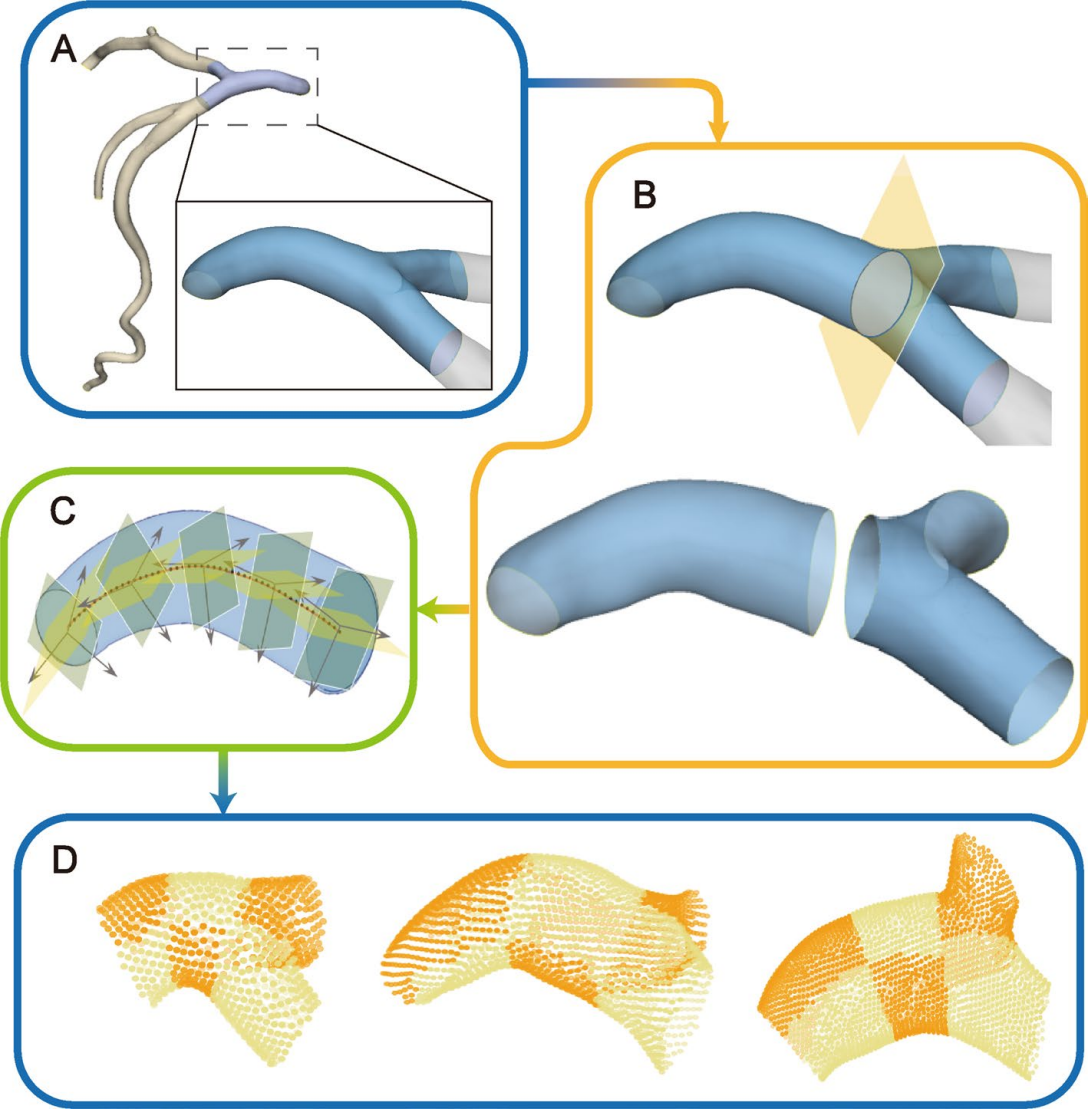
Partitioning of coronary arteries using Frenet–Serret frame. **A** Trim left main (LM) coronary artery and preserve partial left anterior descending artery and left circumflexus. **B** Establish a normal plane through the distal control point of the LM centerline to divide LM into bifurcation and stem segments. **C** Subdivide LM using the normal plane and tangent planes at each control point. **D** Each LM and its branches could be divided into 6 regions

In order to find the variations of L-TAWSS between the different positions of the LM in a more detailed and accurate way, we subdivided the LM and proximal branches by means of a Frenet–Serret frame at each control point of the centerline. Details are contained in Additional file 2: Method and Fig. 6.

**Fig. 7:**
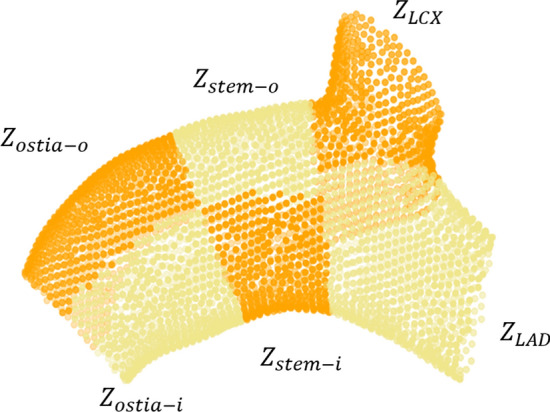
Diagram of LM partition. ${\rm Z}_{{{\text{ostia}} - o}}$, the outer curvature of the ostium part; ${\rm Z}_{{{\text{ostia}} - i}}$, the inner curvature of the ostium part; ${\rm Z}_{{{\text{stem}} - o}}$, the outer curvature of the stem part; ${\rm Z}_{{{\text{stem}} - i}}$, the inner curvature of the stem; ${\rm Z}_{{{\text{LAD}}}}$, the LAD side of bifurcation; ${\rm Z}_{{{\text{LCX}}}}$, the LCX side of bifurcation

The LM was divided into six regions in order to effectively compare the variation in hemodynamic distributions on various LMs. Based on the morphology of the LM’s centerline, the main stem segment was divided into sides with outer and inner curvature and then bisected from the middle. And four regions were identified: the outer curvature of the ostium part (${\rm Z}_{{{\text{ostia}} - o}}$), the inner curvature of the ostium part (${\rm Z}_{{{\text{ostia}} - i}}$), the outer curvature of the stem part (${\rm Z}_{{{\text{stem}} - o}}$), and the inner curvature of the stem (${\rm Z}_{{{\text{stem}} - i}}$). The bifurcation segment was split into the LAD side (${\rm Z}_{{{\text{LAD}}}}$) and LCX side (${\rm Z}_{{{\text{LCX}}}}$) according to the direction of the branches (Fig. 7).

### Statistical analysis

The statistical analysis was conducted using Python (version 3.8) with NumPy, SciPy, and seaborn packages. Categorical variables are characterized by absolute counts and percentages. For continuous variables with a normal distribution, the mean and standard deviation are reported; otherwise, the median and interquartile ranges (IQR) are used. Differences among groups were evaluated using one-way ANOVA (continuous variables with a normal distribution), the Kruskal–Wallis *H* test (continuous variables without a normal distribution), or the *χ*^2^ test (categorical variables). For non-normal continuous variables, the post hoc Dunn test was then utilized to compare two distinct groups. Bonferroni correction was used for the adjustment of multiple comparison. The adjusted *p* value less than 0.05 was deemed statistically significant.

### Supplementary Information

**Additional file 1: Table S1.** Morphological characteristics of the overall LMs and each cluster.

**Additional file 2.** Additional data. Additional materials of methods and results.

## Data Availability

The data that support the findings of this study are available from *Sun Yat-sen Memorial Hospital of Sun Yat-sen University*, but restrictions apply to the availability of these data, which were used under license for the current study, and so are not publicly available. Data are, however, available from the authors upon reasonable request and with permission of *Sun Yat-sen Memorial Hospital of Sun Yat-sen University*.

## Abbreviations

CFD: Computational fluid dynamics
LAD: Left anterior descending artery
LCX: Left circumflex artery
LM: Left main coronary artery
RI: Ramus intermedius artery
TAWSS: Time-averaged wall shear stress
UC: Unsupervised clustering
WSS: Wall shear stress
