# Supplementary material for: Left main coronary artery morphological phenotypes and its hemodynamic properties
Source: Biomed Eng Online. 2024 Jan 22;23:9. doi: 10.1186/s12938-024-01205-3 (PMC10804578; doi:10.1186/s12938-024-01205-3)
Supplement: Supplementary file 1 — Additional file 1: Table S1. Morphological characteristics of the overall LMs and each cluster. [file 12938_2024_1205_MOESM1_ESM.docx]

|  | Total | Cluster 1 | Cluster 2 | Cluster 3 | Cluster 4 | *p* |
| --- | --- | --- | --- | --- | --- | --- |
| $\alpha_{LM-LAD}$,° | 38.02(33.79-46.18) | 43.37(34.07-54.07) | 37.84(35.56-42.41) | 39.81(34.72-50.34) | 30.94(22.46-36.44) | 0.005* |
| $\alpha_{LM-LCX}$,° | 59.02(48.4-67.62) | 46.76(34.56-55.59) | 65.88(61.84-76.78) | 68.05(59.04-72.51) | 55.25(47.18-64.69) | <0.001* |
| $\alpha_{LAD-LCX}$,° | 85.94(74.45-98.62) | 74.09(66.52-80.93) | 100.46(92.73-106.30) | 94.99(91.87-113.32) | 77.72(70.62-81.21) | <0.001* |
| $\alpha_{\mathrm{ostia}}$,° | 73.45(66.31-80.10) | 74.27(72.27-78.71) | 80.06(73.18-84.18) | 59.8(55.67-69.51) | 59.53(51.2-69.56) | <0.001* |
| $\varphi_{\mathrm{LM}}$, mm | 3.77(3.47-4.06) | 3.77(3.58-4.18) | 3.89(3.55-4.24) | 3.63(3.31-3.74) | 3.55(3.24-4.10) | 0.151 |
| $\varphi_{\mathrm{LAD}}/\varphi_{\mathrm{LM}}$ | 0.81(0.75-0.89) | 0.78(0.69-0.84) | 0.79(0.75-0.91) | 0.85(0.82-0.90) | 0.89(0.78-0.92) | 0.006* |
| $\varphi_{\mathrm{LCX}}/\varphi_{\mathrm{LM}}$ | 0.81(0.71-0.90) | 0.75(0.68-0.84) | 0.85(0.78-0.92) | 0.89(0.81-1.10) | 0.79(0.71-0.85) | 0.004* |
| $L_{\mathrm{LM}}$, mm | 9.24(6.43-11.79) | 6.33(4.94-8.65) | 9.83(7.76-12.51) | 11.66(10.15-16.06) | 10.82(7.22-13.33) | <0.001* |
| $\tau_{\mathrm{LM}}$ | 0.03(0.02-0.04) | 0.02(0.01-0.04) | 0.02(0.01-0.04) | 0.08(0.06-0.10) | 0.03(0.02-0.04) | <0.001* |

**Table S1.** Morphological characteristics of the overall LMs and each cluster.

* There are statistical differences between clusters.
