# Supplementary material for: Left main coronary artery morphological phenotypes and its hemodynamic properties
Source: Biomed Eng Online. 2024 Jan 22;23:9. doi: 10.1186/s12938-024-01205-3 (PMC10804578; doi:10.1186/s12938-024-01205-3)
Supplement: Supplementary file 2 — Additional file 2. Additional data. Additional materials of methods and results. [file 12938_2024_1205_MOESM2_ESM.docx]

**Additional Data**

**Additional Method**

1. **Computational fluid dynamics (CFD) analysis of coronary arteries**

**1.1 Mesh Independent Testing**

Mesh generation procedure was performed by Ansys ICEM CFD (version 2022 R1, Ansys, Canonsburg, Pennsylvania). After obtaining the mesh of arteries, we conduct a grid independent test to demonstrate the independence of grid number and time steps from simulation outcomes. To run independent tests, we chose a reconstructed left coronary artery model at random and assigned it a set of meshing settings. Furthermore, the number, shape, layers, and thickness of the prism layer meshes in each model were kept constant in the mesh independence test to prevent the influence of changes in the prism layer meshes on the wall shear stress. For varied grid numbers and time steps, we calculated the average wall shear stress (WSS) in the plane of midway. We assumed that the WSS calculation result was independent of grid number when the difference was less than 1%.

**1.2 Computational fluid dynamics (CFD) analysis**

The Navier-Stokes conservation equation (E1) and the continuity equation (E2) with finite volume approximation are solved by Ansys CFX (version 2022 R1, Ansys, Canonsburg, Pennsylvania):

$$\begin{aligned} \frac{\partial u}{\partial t}+u\cdot\nabla u=-\frac{\nabla P}{\rho}+\nu\nabla^{2}u\#\left（ E1 \right） \end{aligned}$$

$$\begin{aligned} \nabla\cdot u=0\#\left（ E2 \right） \end{aligned}$$

where u is the fluid velocity vector, *P* is the fluid pressure, *ρ* is the fluid density, $\nu$ is the kinematic viscosity, and ∇2 is the Laplacian operator.

The calculation formulas of wall shear stress (WSS) and TAWSS are as follows:

$$\begin{aligned} WSS=\mu\frac{\partial u}{\partial n}\#\left( E3 \right) \end{aligned}$$

$$\begin{aligned} TAWSS=\frac{1}{T}\int_{0}^{T} \left| WSS \right|dt\#\left( E4 \right) \end{aligned}$$

where $\mu$ is dynamic viscosity at 1060 kg/m^3^.

**1.3 Partitioning of coronary arteries**

Splitting the LM and its partial branches before measuring the geometric parameters. Preserve the length of one diameter of LAD and LCX from the starting control point of the centerline (**Figure 6A**). A normal plane was first established through the control point at the distal end of the centerline to divide the LM into the bifurcation segment and the main stem segment (**Figure 6B**).

The partitioning of the coronary arteries was accomplished by combining the centerline extracted from the coronary arteries with the Frenet-Serret frame. A Frenet-Serret frame can be constructed for each point on a spatial curve. It consists of three mutually perpendicular unit vectors, which are the tangent vector $T\left（ t \right）$, the normal vector $N(t)$ and the subnormal vector $B(t)$ of this point. Where $t$ denotes the arc length on the curve. $T\left（ t \right）$ is a unit vector tangent to the space curve, pointing to the motion direction of the point on the curve. $N(t)$ is a unit vector perpendicular to the tangential vector and points in the direction of the centripetal acceleration of the curve. $B(t)$ is a unit vector perpendicular to both the tangent vector and the normal vector, which points in the direction of their cross product. Where the plane formed by $T\left（ t \right）$ and $N(t)$ is called the osculating plane, $N(t)$ and $B(t)$ form the normal plane, and $T\left（ t \right）$ and $B(t)$ form the rectifying plane.

Each control point $C$ on the centerline measured by the Mimics software contains the coordinates of the point ($x_{c} ,y_{c} , z_{c}$), the tangent vector ($x_{\tau} ,y_{\tau} , z_{\tau}$), the normal vector ($x_{\nu} ,y_{\nu} , z_{\nu}$), the subnormal vector ($x_{\beta} ,y_{\beta} , z_{\beta}$) and other features of the spatial curve over the control point. With the above vectors and control point coordinates, the equations for each plane can be derived as follows:

For a point $P\left（ a,b,c \right）$, if it is on the normal plane, the defining equation of normal plane is:

$$\begin{aligned} a\left( x_{\tau}-x_{c} \right)+b\left( y_{\tau}-y_{c} \right)+c\left( z_{\tau}-z_{c} \right)=0\#\left( E5 \right) \end{aligned}$$

if $P$ is on the osculating plane, define the equation for osculating plane is:

$$\begin{aligned} a\left( x_{\beta}-x_{c} \right)+b\left( y_{\beta}-y_{c} \right)+c\left( z_{\beta}-z_{c} \right)=0\#\left( E6 \right) \end{aligned}$$

if $P$ is on the rectifying plane, the equation of the rectifying plane is:

$$\begin{aligned} a\left( x_{\nu}-x_{c} \right)+b\left( y_{\nu}-y_{c} \right)+c\left( z_{\nu}-z_{c} \right)=0\#\left( E7 \right) \end{aligned}$$

A 3D coordinate plane is constructed through a coordinate system and three vectors at control points. Each coordinate plane can divide the 3D coronary artery into two parts at the corresponding control point. The LM is divided into upper and lower segments along the longitudinal axis through the normal plane of each control point, and the coronary artery between every two adjacent normal planes is defined as a subdivided coronary artery layer. Then each layer can be further subdivided into inner and outer parts through the tangential plane at each control point (**Figure 6C**). The segmented LM layers and their inner and outer subdivisions are further divided into four regions: proximal inner and outer regions at the LM ostium, and inner and outer regions at the stem of LM, denoted as $Z_{ostia-i}$, $Z_{ostia-o}$, $Z_{stem-i}$ and $Z_{stem-o}$, respectively. Using the first centroid of the proximal centerline as the center, and the diameter of each tangential circle as the radius, a portion of the coronary artery is segmented and preserved for both LAD and LCX, denoted as $Z_{LAD}$ and $Z_{LCX}$, respectively. The LM segmentation pattern is shown in **Figure 6**.

All the aforementioned steps were completed using Python 3.8, with the help of packages such as numpy, pandas, matplotlib, etc. With these steps, we can segment the CFD node coordinates and indicators of each subregion, which can be used for subsequent statistical analyses.

1. **Result**

**2.1 Study population**

Retrospective selection of patients who underwent CCTA and without significant coronary atherosclerotic plaques at Sun Yat-sen Memorial Hospital of Sun Yat-sen University from March 2019 to May 2019. Excluding 19 patients with a history of cardiac surgery, 8 with severe image artifacts, 26 with structural heart diseases such as heart valve disease and cardiomyopathy, and 9 with severely compressed myocardial bridges. A total of 96 patients were included, including 48 (50%) males. 96 LCAs were reconstructed in three dimensions, excluding 4 arteries with unsatisfactory quality after reconstruction, 3 with incomplete centerline data, 1 with coronary artery malformation such as arteriovenous fistula, and 12 with the RM. Finally, 76 LCAs were included, of which 37 (48.7%) were from male. Baseline Characteristics for all enrolled patients and for each group is shown in Table S2.

|  | Total,  n=76 | Cluster 1, n=26 | Cluster 2, n=26 | Cluster 3, n=10 | Cluster 4, n=14 | *p* |
| --- | --- | --- | --- | --- | --- | --- |
| Age, years | 57.21±11.54 | 60.54±13.15 | 55.27±10.78 | 60.40±10.44 | 52.36±8.67 | 0.103 |
| Male, (%) | 37(48.7%) | 11(42.3%) | 14(53.8%) | 6(60%) | 6(42.9%) | 0.705 |
| BMI, Kg/m^2^ | 22.01±2.86 | 22.21±2.91 | 24.04±2.75 | 22.84±2.14 | 22.66±3.10 | 0.128 |
| Hypertension, (%) | 27(35.5%) | 9(34.6%) | 9(34.6%) | 4(40%) | 5(35.7%) | 0.991 |
| Diabates, (%) | 20(26.7%) | 6(24%) | 7(26.9%) | 3(30%) | 4(28.6%) | 0.982 |
| Hyperlipemia, (%) | 35(46.7%) | 11(44%) | 10(38.5%) | 8(80%) | 6(42.9%) | 0.150 |

**Table S2.** Clinical data of the overall population and four clusters.

BMI, Body Mass Index.

**Figures Legends**

**
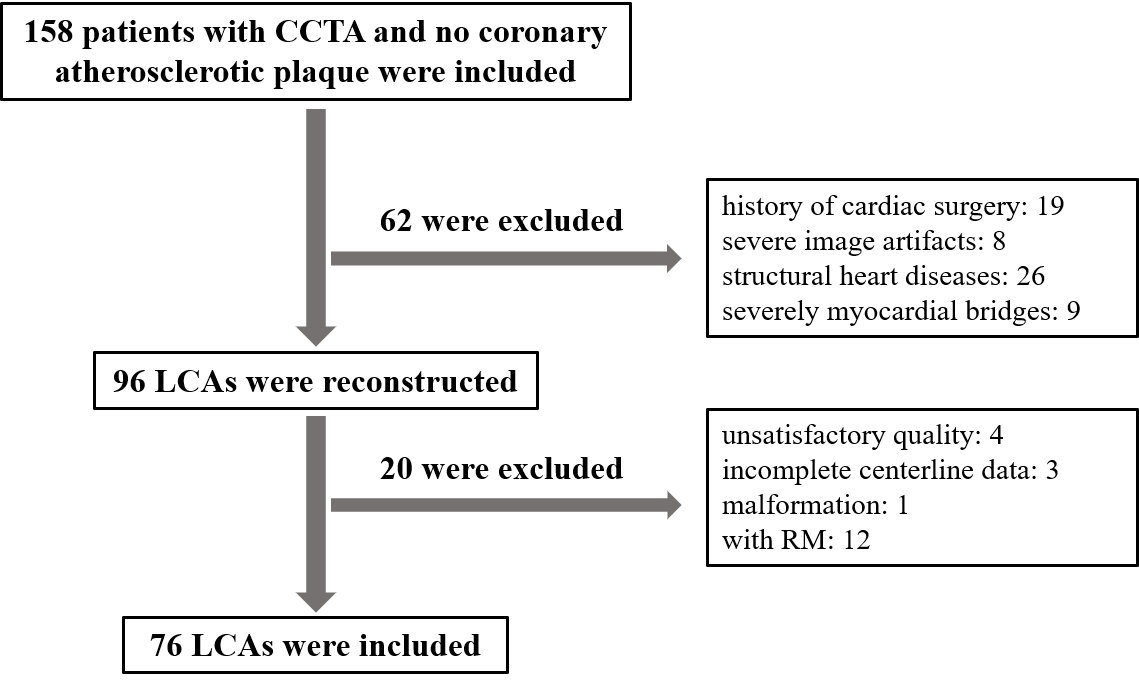
Figure S1**. Inclusion and exclusion of the patients. CCTA, Coronary CT Angiography; LCAs, Left Coronary Arteries.


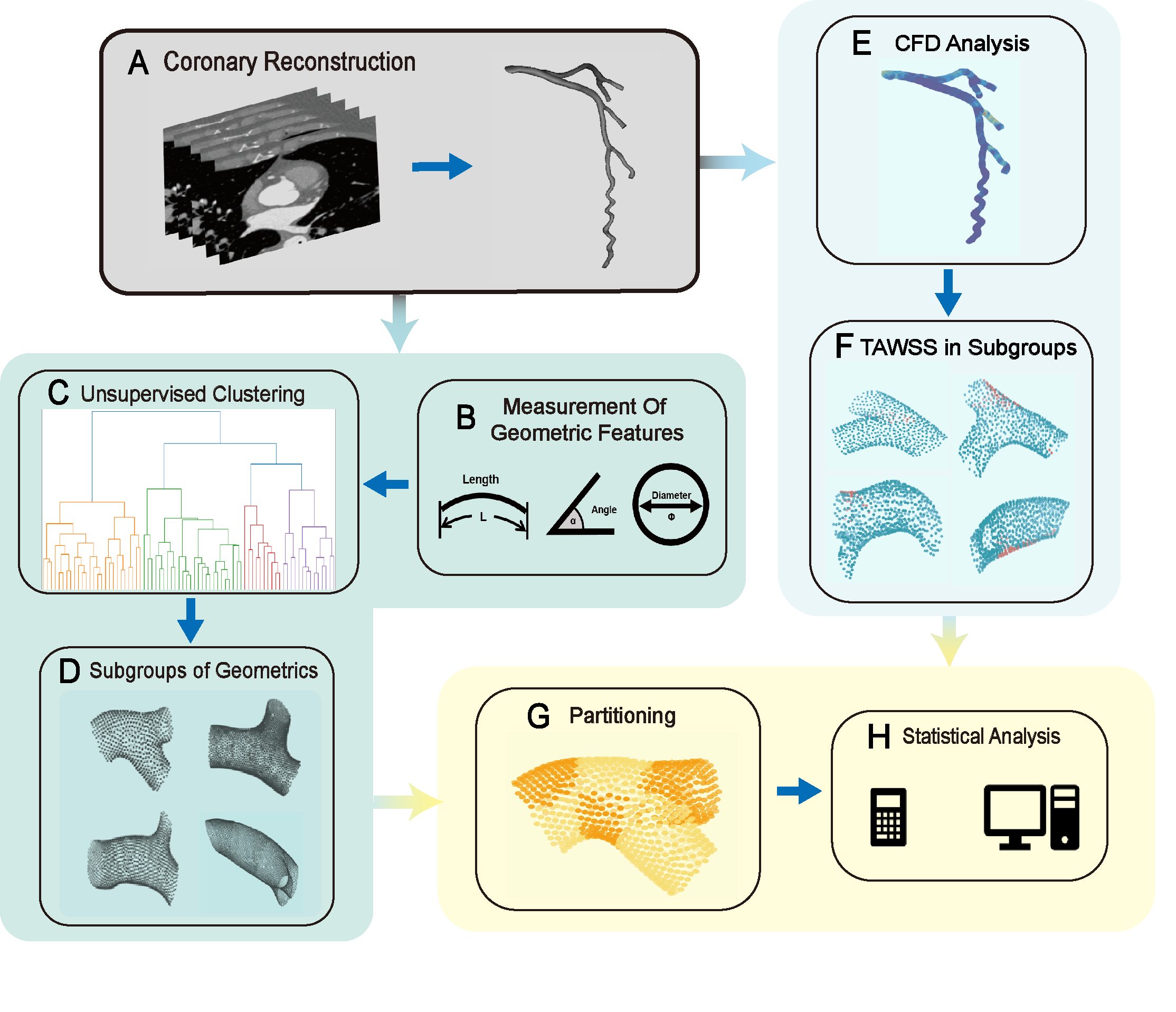
**Figure S2** The flow chart of the study. After reconstruction of the left coronary artery and its branches from coronary computed tomography angiography (A), the geometric parameters of the left main (LM) coronary artery were measured (B). Morphological characteristics of the LM were clustered by hierarchical clustering and 4 phenotypes were observed (C and D). Computational fluid dynamics analysis was performed on the reconstructed coronary arteries (E). Analyze the distribution of time-averaged wall shear stress (TAWSS) nodes in LM (F). Every LM was divided into 6 regions (G), which were used to analyze the distribution of TAWSS nodes in different regions of each LM phenotype (H).
